# Negative potential energy content analysis in cracked rotors whirl response

**DOI:** 10.1038/s41598-021-94836-8

**Published:** 2021-07-27

**Authors:** Mohammad A. AL-Shudeifat, Fatima K. Alhammadi

**Affiliations:** 1 Khalifa University of Science & Technology, PO BOX 127788, Abu Dhabi, United Arab Emirates; 2 Tawazun Technology & Innovation, PO BOX 908, Abu Dhabi, United Arab Emirates

**Keywords:** Aerospace engineering, Mechanical engineering, Engineering

## Abstract

Appearance of transverse cracks in rotor systems mainly affects their stiffness content. The stability of such systems at steady-state running is usually analyzed by using the Floquet’s theory. Accordingly, the instability zones of rotational speeds are dominated by negative stiffness content in the whirl response in the vicinity of critical rotational speeds. Consequently, an effective stiffness measure is introduced here to analyze the effect of the crack and the unbalance force vector orientation on the intensity of negative potential and stiffness content in the whirl response. The effective stiffness expression is obtained from the direct integration of the equations of motion of the considered cracked rotor system. The proposed effective stiffness measure is applied for steady-state and transient operations using the Jeffcott rotor model with open and breathing crack models. The intensity of negative potential and stiffness content in the numerical and experimental whirl responses is found to be critically depending on the propagation level of the crack and the unbalance force vector orientation. Therefore, this can be proposed as a crack detection tool in cracked rotor systems that either exhibit recurrent passage through the critical rotational speeds or steady-state running.

## Introduction

Appearance of transverse cracks in rotor systems significantly affects their whirl response dynamical behavior. Propagation of transverse cracks could result in destructive whirl vibration amplitudes and rapid failure of rotor systems. Therefore, early detection of crack damages is critical for human safety and durability of rotor systems that are extensively applied in heavy-duty industrial applications.

Several types of crack damages have been addressed in the literature. However, two main types of cracks which are the transverse open and breathing crack models, have been extensively studied up-to-date. Most of the reported literature for transient and steady-state operations of rotor systems have been focused on the dynamical behavior of vibration whirl response in the vicinity of critical, subcritical and supercritical rotational speeds. The whirl dynamical behavior in the vicinity of critical and subcritical rotational speeds was investigated in^1^ to differentiate between the whirl responses of these crack models in a cracked rotor system at steady-state operations. It was found that the forward subcritical whirl amplitudes were not observed in open crack model compared with breathing crack model. Several Vibration-Based Methods (VBMs) have been employed for crack damage detection. In^2–5^, the VBMs were used for damage detection in rotor systems through whirl response analysis. In addition, VBMs have been applied to study the effect of crack damages on stiffness and damping properties in^6,7^. Analytical and numerical methods were applied to the equations of motion of a cracked rotor system in^8^ to study the whirl response dynamical behavior.

In several studies, the transient whirl response during startup operations in which angular acceleration rate was considered in the equations of motion has been well-studied for cracked rotor systems with open and breathing crack models in^9–16^. In^9^, experimental investigations have been conducted for analyzing the transient vibrational whirl behavior during the passage through the critical rotational speed. The transient whirl response was investigated to distinguish between the effect of the transverse and slant crack models in^10,11^ and between the responses of transverse crack and the shaft misalignment in rotor systems in^12^. Recently, a new backward whirl phenomena named as a post-resonance backward whirl has been captured in the transient response of accelerated cracked rotor systems immediately after the passage through the critical forward whirl speeds^13–15^. This post-resonance backward whirl was found to be associated with an abrupt reduction in whirl amplitudes in the vicinity of local minima of whirl amplitudes. The Hilbert–Huang Transform (HHT) which is a time frequency analysis method was employed in^16,17^ to study the transient vibration whirl response of rotors with transverse cracks.

In another series of publications, several models of cracked rotor systems were employed with several damage detection techniques for studying the vibration whirl signature of cracked rotor systems at steady-state running^18–25^. Modal analysis was used in^18^ to detect the variations in natural frequencies, mode shapes, and the response to an applied forcing excitation in cracked rotors. Moreover, this method was used for investigating the effect of position and depth of the transverse crack on the modal properties of the considered systems. In^19,20^, a coupled radial, axial and torsional vibration whirl response was analyzed for crack detection and identification. In^21^, a static rotor system with an open crack model in the shaft was considered for vibration whirl analysis to predict the transverse crack depth and its location. Different excitation kinds have been applied with rotor systems in^22–24^ including multi-sine, parametric and mixed excitations. The multi-sine excitation method was used to evaluate the effect of the crack on nonlinear distortions in the whirl response of a cracked rotor system in^22^. The multiple scale method was applied to the equations of motion of rotors with active magnetic bearings in^23^ and with both active magnetic bearings and time varying stiffness in^24^ for stability analysis at steady-state solution. Moreover, an effective time-domain identification algorithm based on the Extended Kalman Filter (EKF) was employed in^25^ for rotor damage detection where the neural networks accompanied with power spectral density characterization have been applied in^26^ for crack damage identification.

The flexibility matrix and finite element methods were employed in determining the time-periodic stiffness matrix of cracked rotor systems in several publications such as in^1,7,9^ and in^27–29^. In some other studies, the stability of the cracked rotor system was addressed. The Jeffcott rotor with a breathing crack model was considered in^30^ and the FE model in^31^ for determining the effect of the parametric excitation caused by the time-varying stiffness on the stability of cracked rotor systems. The unstable zones are usually obtained by applying the Floquet’s theory to the free response of the parametrically excited cracked rotor system where the state-transition matrix is formulated using an identity matrix of initial conditions. The obtained unstable zones from the eigensolution of the state-transition matrix are associated with negative potential and stiffness energy content.

In recent series of publications, different methods and techniques have been applied for fault diagnostics in rotor systems. The autonomous ultrasonic testing method was employed in^32^ for monitoring crack initiation and propagation by collecting the data of real-time whirl response using an ultrasonic wave system. In addition, signal processing methods were applied in^33,34^ to cracked rotors whirl response. Therefore, the auto-correlation and power spectral density functions were employed in^33^ and the time–frequency info-grams method was employed in^34^. The robustness of both methods was also verified by experimental results. Furthermore, the non-linear output frequency response function NOFRF was used for damage diagnostics in rotor systems in^35,36^ where numerical simulation and experimental testing have been employed. In^35^, the weighted rate-based NOFRF was applied with special index for rotor crack detection. This method was numerically and experimentally validated where the crack detection index has been observed to linearly proportional to the crack depth. However, further analysis was performed using the NOFRF in^36^ to identify the crack depth and its location by using a crack position index (CPI) based on the higher harmonic response (HHR) and the dynamic compliance matrix. Another method based on fracture-mechanic principles has been applied in^37^. This method depends on the change in compliance matrix that induced by crack propagation in the shaft of steam turbine. Accordingly, the changes in natural bending, longitudinal, and torsional frequencies have been employed as an indication to cracks propagation. A different multi-harmonic based technique was addressed in^38^ for crack detection diagnostics. An impedance-based structural health monitoring (ISHM) was studied in^39,40^. The detection of a crack propagation in rotating shafts was performed by employing the real-time impedance-based structural health monitoring method in^39^. This method is based on using piezoelectric transducers to perform a compact function of fault sensing and responsive signal actuation. In^40^, the comparison results between the sensitivity matrices of single and double piezoelectric transducers was used as rotor damage indicator. The Discrete Wavelet Transform (DWT) method which is followed by auto-correlation process, was found to be effective for rotor damage detection in^41^.

In the reviews of the state-of-the-art in^42–44^, several crack detection methods and cracked rotor modeling techniques have been addressed where different kinds of cracked rotor models and several methods of whirl response analysis have been reviewed and discussed. The review performed in^42^ has addressed the literature that focused on the effect of induced cracked-based nonlinearities on sub and super harmonics components in the whirl response. The use of variety of signal-processing methods as tools for differentiating between crack-based nonlinearities from other kinds of nonlinearities in rotors was also addressed in that review. A list of most common methodologies of studying cracked rotors in various publications was provided and discussed in^43^. In another review, the artificial intelligence-based rotor fault diagnostic (AI-RFD) was reviewed in^44^ where the signal-processing based techniques were discussed and summarized.

In this study, an effective stiffness measure is introduced based on our prior study in^45^ and applied to cracked rotor systems. Therefore, the impact of both open and breathing crack models at steady-state and transient running on the appearance of high negative potential and stiffness zones of rotational speeds is investigated. In addition, the influence of the unbalance force vector orientation on the negative potential content is considered in the numerical simulations and the experimental validations.

## Cracked rotor modeling

**Figure 1:**
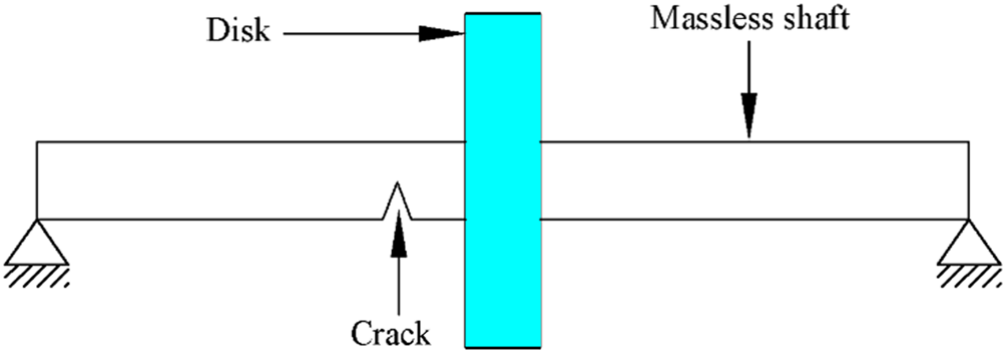
Jeffcott rotor with an open crack model.

The two-degree-of-freedom Jeffcott rotor model is considered here where it is represented by a concentric rigid disk of mass *m* at the mid-span of simply-supported elastic massless shaft as shown in Fig. 1. The equations of motion of the intact Jeffcott rotor model for the vector of horizontal and vertical whirl amplitudes $${\varvec{q}}\left( t \right) = [\begin{array}{*{20}c} {u_{x} \left( t \right)} & {v_{y} \left( t \right)]} \\ \end{array}^{{\text{T}}}$$ and unbalanced force excitation vector ${\varvec{F}}_{u}^{{}} \left( t \right)$ including the gravity effect are given in matrix form as

1 $$ \user2{M\ddot{q}}\left( t \right) + \user2{C\dot{q}}\left( t \right) + {\varvec{Kq}}\left( t \right) = {\varvec{F}}_{u}^{{}} \left( t \right) $$

where ${\varvec{M}}$, ${\varvec{C}}$, and ${\varvec{K}}$ are the mass, damping and stiffness matrices. The matrices ${\varvec{M}}$ and ${\varvec{K}}$, and the unbalance force vector ${\varvec{F}}_{u}^{{}} \left( t \right)$ for steady-state operation of intact Jeffcott rotor system are given, respectively, as

2 $$ {\varvec{M}} = \left[ {\begin{array}{*{20}c} m & 0 \\ 0 & m \\ \end{array} } \right],{\varvec{K}} = \frac{48E}{{L^{3} }}\left[ {\begin{array}{*{20}c} {I_{{}} } & 0 \\ 0 & {I_{{}} } \\ \end{array} } \right]\;{\text{and}}\;{\varvec{F}}_{u}^{{}} \left( t \right) = \left[ {\begin{array}{*{20}c} {m\varepsilon \Omega^{2} \cos \left( {\Omega t} \right)} \\ {m\varepsilon \Omega^{2} \sin \left( {\Omega t} \right) - mg} \\ \end{array} } \right] $$

where *m* is the rigid disk mass, $L$ is the length of the shaft, $E$ is the modulus of elasticity of the shaft, $I$ is the area moment of inertia of the shaft cross-section, $\Omega$ is the shaft angular rotational speed and $\varepsilon$ is the eccentricity of the unbalance force mass.

The appearance of transverse crack in the shaft cross-sections is associated with time-varying stiffness matrix which is represented by ${\varvec{K}}_{C} \left( t \right)$. Therefore, Eq. (1) can be rewritten for a cracked system as

3 $$ \user2{M\ddot{q}}\left( t \right) + \user2{C\dot{q}}\left( t \right) + {\varvec{K}}_{C} \left( t \right){\varvec{q}}\left( t \right) = {\varvec{F}}_{u}^{{}} \left( t \right) $$

### Steady-state operation

Here we consider the open and breathing crack models for studying the potential energy content in a cracked Jeffcott rotor system at steady-state running.

#### Open crack model

**Figure 2:**
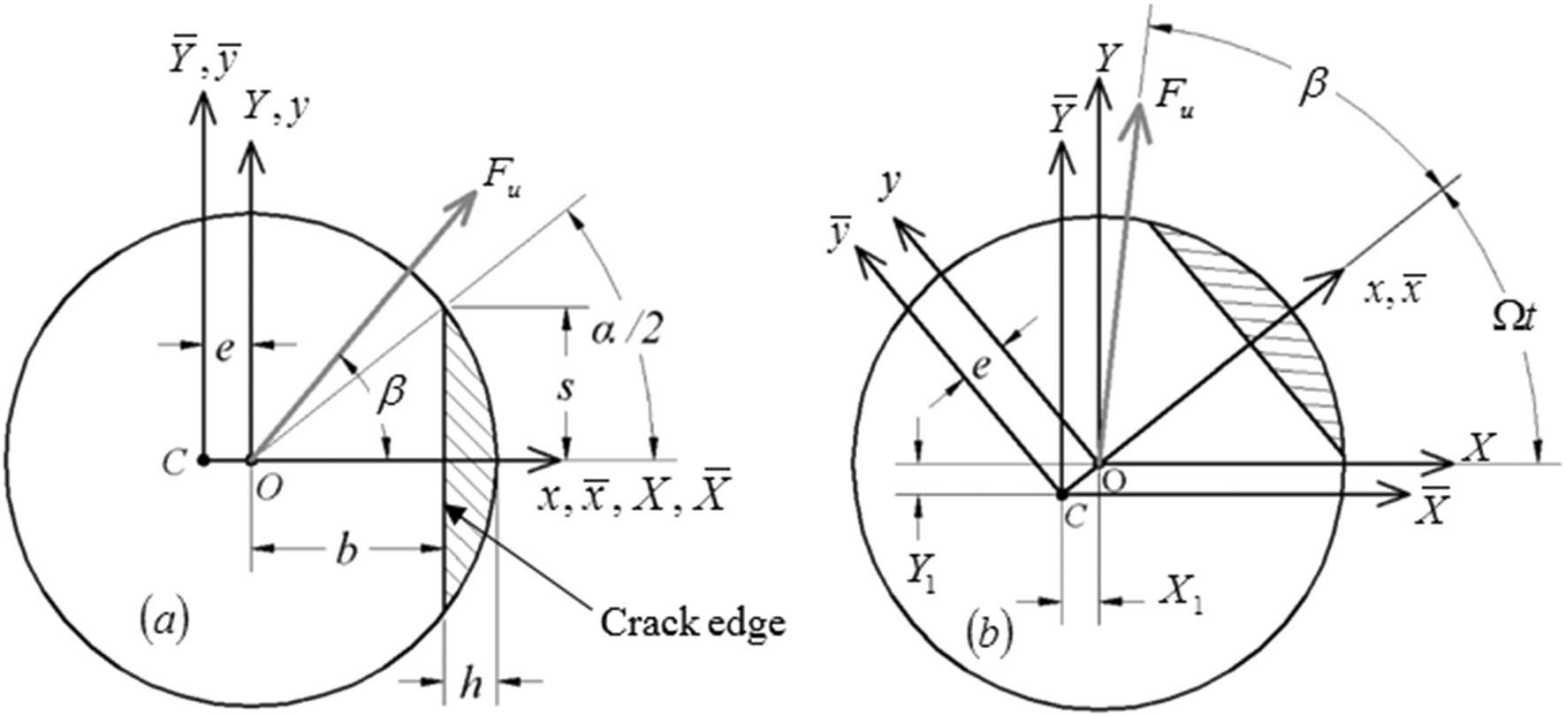
Schematic diagrams of the open crack in the shaft cross-section (**a**) before the shaft’s rotation, and (**b**) after the shaft’s rotation^13–15^.

The transverse open crack model is considered here as shown in Fig. 2. The depth of the crack in the transverse radial direction is expressed by $h$. The crack opening orientation at the beginning of shaft’s rotation is assumed to be at zero angle with respect to the fixed *X* axis. The unbalance force vector orientation with respect to the crack opening direction is represented by the angle $\beta$ as shown.

If the shaft has a transverse open crack as shown in Fig. 2, the stiffness matrix in the rotating centroidal coordinates $\overline{x}$ and $\overline{y}$ axes is written as

4 $$ {\varvec{K}}_{R} = \frac{48E}{{L^{3} }}\left[ {\begin{array}{*{20}c} {I_{{\overline{y}}} } & 0 \\ 0 & {I_{{\overline{x}}} } \\ \end{array} } \right] $$

where $I_{{\overline{y}}} = I_{y} - e^{2} A_{ce}$ and $I_{{\overline{x}}} = I_{x}$ are the area moments of inertia with respect to the centroidal rotating coordinates. The quantities $I_{x}$, $I_{y}$, $A_{ce}$ and *e* are found for the normalized crack depth $\mu = h/R$ in^1,13^. The cracked element stiffness matrix ${\varvec{K}}_{C}^{{}} \left( t \right)$ with respect to the stationary coordinates is obtained by transforming ${\varvec{K}}_{{\text{R}}}^{{}}$ via the following transformation

5 $$ {\varvec{K}}_{C}^{{}} \left( t \right) ={\varvec{\varPsi}}^{{}} {\varvec{K}}_{R}^{{}}{\varvec{\varPsi}}^{{\mathbf{T}}} $$

where ${\varvec{\varPsi}}$ is the coordinate transformation matrix which is expressed as

6 $${\varvec{\varPsi}}= \left[ {\begin{array}{*{20}c} {\cos (\Omega t)} & { - \sin (\Omega t)} \\ {\sin (\Omega t)} & {\cos (\Omega t)} \\ \end{array} } \right] $$

Accordingly, the resultant stiffness matrix ${\varvec{K}}_{C} \left( t \right)$ in the stationary coordinates is obtained from Eq. (5) for the open crack model as

7 $$ {\varvec{K}}_{C} \left( t \right) = {\varvec{K}}_{1}^{{}} + {\varvec{K}}_{2}^{{}} \cos \left( {2\Omega t} \right) + {\varvec{K}}_{3}^{{}} \sin \left( {2\Omega t} \right) $$

where $\Omega$ is the constant angular rotational speed. For $\overline{I}_{1} = \left( {1/2} \right)\left( {I_{{\overline{x}}} + I_{{\overline{y}}} } \right)$ and $\overline{I}_{2} = \left( {1/2} \right)\left( {I_{{\overline{x}}} - I_{{\overline{y}}} } \right)$, the matrices ${\varvec{K}}_{1}^{{}}$, ${\varvec{K}}_{2}^{{}}$ and ${\varvec{K}}_{3}^{{}}$ are obtained from the transformation as

8 $$ {\varvec{K}}_{{\mathbf{1}}} = \frac{48E}{{L^{3} }}\left[ {\begin{array}{*{20}c} {\overline{I}_{1} } & 0 \\ 0 & {\overline{I}_{1} } \\ \end{array} } \right], \, {\varvec{K}}_{{\mathbf{2}}} = \frac{48E}{{L^{3} }}\left[ {\begin{array}{*{20}c} { - \overline{I}_{2} } & 0 \\ 0 & {\overline{I}_{2} } \\ \end{array} } \right],{\text{ and }}{\varvec{K}}_{{\mathbf{3}}} = \frac{48E}{{L^{3} }}\left[ {\begin{array}{*{20}c} 0 & { - \overline{I}_{2} } \\ { - \overline{I}_{2} } & 0 \\ \end{array} } \right] $$

#### Breathing crack model

The breathing crack model in^15,30^ is also considered here to obtain the time-varying stiffness matrix ${\varvec{K}}_{C}^{{}} \left( t \right)$. Therefore, the instantaneous time-varying moments of area in the cracked cross-section are firstly calculated based on the shaft angle of rotation $\theta \left( t \right) = \Omega t$ at each time step in the numerical integration as

9a $$ I_{{\overline{X}}} \left( t \right) = I - \left( {I - \overline{I}_{1} } \right)f_{1} \left( t \right) $$

9b $$ I_{{\overline{Y}}} \left( t \right) = I + \left( {I - \overline{I}_{1} } \right)f_{1} \left( t \right) - \left( {2I - \overline{I}_{1} - \overline{I}_{2} } \right)f_{2} \left( t \right) $$

9c $$ I_{{\overline{X}\overline{Y}}} \left( t \right) = \left( { - \frac{{\overline{I}_{1} - \overline{I}_{2} }}{2} + \frac{{A_{1} e^{2} }}{2}} \right)\sin \left( {2\theta \left( t \right)} \right)\sum\limits_{k = 1}^{p} {\frac{{2\overline{\theta }_{2} \sin \left( {\pi - k\overline{\theta }_{2} } \right)}}{{\pi^{2} - k^{2} \overline{\theta }_{2}^{2} }}} \sin \left( {k\theta \left( t \right)} \right) $$

where $f_{1} \left( t \right)$ and $f_{2} \left( t \right)$ are the crack breathing functions which are given as

10a $$ f_{1} \left( t \right) = \cos^{n} \left( {\frac{\theta \left( t \right)}{2}} \right) = \frac{1}{{2^{n} }}\left[ {\left( {\begin{array}{*{20}c} n \\ {n/2} \\ \end{array} } \right) + 2\sum\limits_{j = 0}^{{\left( {{n \mathord{\left/ {\vphantom {n 2}} \right. \kern-\nulldelimiterspace} 2}} \right) - 1}} {\left( {\begin{array}{*{20}c} n \\ j \\ \end{array} } \right)\cos \left( {\left( {n - 2j} \right)\frac{\theta \left( t \right)}{2}} \right)} } \right] $$

10b $$ f_{2} \left( t \right) = \frac{1}{\pi }\left[ {\frac{{\theta_{1} + \theta_{2} }}{2} - \frac{2}{{\left( {\theta_{2} - \theta_{1} } \right)}}\sum\limits_{i = 1}^{p} {\frac{{\cos \left( {i\theta_{2} } \right) - \cos \left( {i\theta_{1} } \right)}}{{i^{2} }}\cos \left( {i\theta \left( t \right)} \right)} } \right] $$

where $\theta_{1}$ is the angles at which the crack starts to close and $\theta_{2}$ is the angle at which the crack becomes fully close. The formulas of $\theta_{1}$, $\theta_{2}$, $I$, $\overline{I}_{1}$, $\overline{I}_{2}$, $A_{1}$ and $e$ are found in^15,30^ and provided in the appendix. For crack depths near to $\mu = 0.2$ or below this value, the values of $p$ and $n$ in Eqs. (9) and (10) are selected to be equal to 16 and 12, respectively, to guarantee a sufficient convergence of the approximate values of moments of area to the exact values in the cracked cross-section. Accordingly, the cracked shaft stiffness matrix ${\varvec{K}}_{C}^{{}} \left( t \right)$ in the fixed centroidal coordinates for a breathing crack model is now expressed as

11 $$ {\varvec{K}}_{C}^{{}} \left( t \right) = \left[ {\begin{array}{*{20}c} {K_{{\overline{Y}}} (t)} & {K_{{\overline{X}\overline{Y}}} (t)} \\ {K_{{\overline{X}\overline{Y}}} (t)} & {K_{{\overline{X}}} (t)} \\ \end{array} } \right] = \frac{48E}{{L^{3} }}\left[ {\begin{array}{*{20}c} {I_{{\overline{Y}}} (t)} & {I_{{\overline{X}\overline{Y}}} (t)} \\ {I_{{\overline{X}\overline{Y}}} (t)} & {I_{{\overline{X}}} (t)} \\ \end{array} } \right] $$

### Transient operation

#### Open crack model

For transient operation, the angular acceleration rate during the shaft rotation significantly alters the system’s mathematical model. The internal material damping is also considered according to^46^ to be proportional to the stiffness matrix ${\varvec{K}}$ in Eq. (2) of the intact shaft. Accordingly, this damping content is expressed as ${\varvec{D}}_{R} = \zeta {\varvec{K}}$ where $\zeta$ is the internal viscous damping coefficient. Therefore, the internal damping matrix ${\varvec{D}}_{R}$ is transformed into the fixed coordinates using the transformation matrix ${\varvec{\varPsi}}$ which was previously given in Eq. (6) to obtain the damping force in the fixed coordinates as ${\varvec{F}}_{D} = - \zeta \left( {\user2{K\dot{q}}\left( t \right) + {\varvec{K}}_{cir} {\varvec{q}}\left( t \right)} \right)$ where ${\varvec{K}}_{cir}$ is the skew-symmetric circulation matrix which is given as

12 $$ {\varvec{K}}_{cir} = \frac{48E}{{L^{3} }}\left[ {\begin{array}{*{20}c} 0 & { - I} \\ I & 0 \\ \end{array} } \right] $$

Accordingly, for the angle of rotation $\theta \left( t \right) = 0.5\alpha t^{2}$ and angular speed $\Omega \left( t \right) = \alpha t$ at the angular acceleration rate $\alpha$, the stiffness matrix ${\varvec{K}}_{C} \left( t \right)$ in Eq. (3) of the cracked shaft with an open crack model and the damping matrix ${\varvec{C}}$ for transient rotation are expressed as

13 $$ \begin{aligned} & {\varvec{K}}_{C} \left( t \right) = {\varvec{K}}_{1}^{{}} + {\varvec{K}}_{2}^{{}} \cos \left( {2\theta \left( t \right)} \right) + {\varvec{K}}_{3}^{{}} \sin \left( {2\theta \left( t \right)} \right) + \Omega (t)\zeta {\varvec{K}}_{cir} \\ & {\varvec{C}} = \gamma {\varvec{M}} + \zeta {\varvec{K}}_{R} \\ \end{aligned} $$

where $\gamma$ is the external viscous damping coefficient which is selected to be $\gamma = 100{\text{ s}}^{ - 1}$ according to^46^. In addition, $\zeta$ was usually assumed of order $O(10^{ - 7} )$ for steel shafts in the literature^47,48^. Therefore, $\zeta$ is selected for the numerical simulation as $\zeta = 2 \times 10^{ - 7} {\text{ s}}$.

#### Breathing crack models

Considering the breathing crack model, the stiffness matrix ${\varvec{K}}_{C}^{{}} \left( t \right)$ of the cracked shaft is still obtained according to Eqs. (9–11) at $\theta \left( t \right) = 0.5\alpha t^{2}$ and $\Omega \left( t \right) = \alpha t$. Moreover, the term including the circulation matrix in Eq. (12) should be also maintained in the total stiffness of the cracked shaft with breathing crack model where the total stiffness becomes ${\varvec{K}}_{C}^{{}} \left( t \right) + \Omega (t)\zeta {\varvec{K}}_{cir}$ rather than ${\varvec{K}}_{C}^{{}} \left( t \right)$ in Eq. (3).

During transient operations, the angular acceleration rate $\alpha$ is also affecting the unbalance force vector components for both open and breathing crack models. Accordingly, by incorporating the angular acceleration rate and the unbalance force vector angle $\beta$ of the cracked shaft, the unbalance force vector ${\varvec{F}}_{u}^{{}} \left( t \right)$ including the gravity effect at $\theta \left( t \right) = 0.5\alpha t^{2}$ and $\Omega \left( t \right) = \alpha t$ is written as

14 $$ {\varvec{F}}_{u} \left( t \right) = \left[ {\begin{array}{*{20}c} {m\varepsilon \alpha^{2} t^{2} \cos (\alpha t^{2} /2 + \beta ) + m\varepsilon \alpha \sin \left( {\alpha t^{2} /2 + \beta } \right)} \\ {m\varepsilon \alpha^{2} t^{2} \sin (\alpha t^{2} /2 + \beta ) - m\varepsilon \alpha \cos \left( {\alpha t^{2} /2 + \beta } \right) - mg} \\ \end{array} } \right] $$

## Effective stiffness calculations

Here, an effective stiffness measure for the considered cracked Jeffcott rotor system at steady-state and transient operations is introduced based on^45^. By pre-multiplying the equations of motion in Eq. (3) by $\dot{\user2{q}}^{T}$ and integrating for zero initial condition from $t = 0$ to the steady-state time of operation $t_{ss}$, we obtain

15 $$ \frac{1}{2} \, \dot{\user2{q}}^{T} \user2{M\dot{q}} + \int\limits_{0}^{{t_{ss} }} {\dot{\user2{q}}^{T} \user2{C\dot{q}} \, dt} + \int\limits_{0}^{{t_{ss} }} {\dot{\user2{q}}^{T} {\varvec{K}}_{C}^{{}} \left( t \right){\varvec{q}} \, dt} = \int\limits_{0}^{{t_{ss} }} {\dot{\user2{q}}^{T} {\varvec{F}}_{u} (t)dt} $$

Therefore, from this equation we obtain the following representation of the total potential energy in the cracked rotor system as

16 $$ P\left( t \right) = \int\limits_{0}^{t} {\dot{\user2{q}}^{T} {\varvec{K}}_{C} \left( t \right){\varvec{q}} \, dt} $$

This potential energy in Eq. (14) can be written in the following equivalent form as

17 $$ P\left( t \right) = \frac{1}{2}k_{eff} \left( t \right)z\left( t \right)_{{}}^{2} $$

where $z\left( t \right)$ is the whirl amplitude which is obtained from $z\left( t \right) = \sqrt {u_{x} \left( t \right)^{2} + v_{y} \left( t \right)^{2} }$. Accordingly, the effective stiffness content is obtained as

18 $$ k_{eff} \left( t \right) = \frac{2P\left( t \right)}{{z\left( t \right)_{{}}^{2} }} $$

## Numerical simulation and experimental results

**Figure 3:**
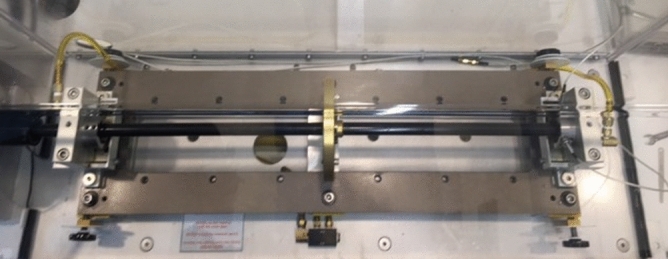
Spectra Quest MFS-RDS rotordynamic simulator.

The physical parameters in Table 1 of the Spectra Quest MFS-RDS lab rotordynamic simulator shown in Fig. 3 are employed in the numerical simulation to obtain the potential energy and effective stiffness measures based on Eqs. (16) and (18). This simulator is employed for experimental validation of the numerical simulation results where it incorporates a single rigid disk at the mid-span of the shaft as shown. Two proximity probes were installed near to the rigid disk to collect the experimental data of the horizontal and vertical whirl amplitudes of the system. In addition, the instantaneous shaft rotational speed is automatically collected at the same proximity probes frequency which is equivalent to 5 kHz. The collected whirl response vector by the proximity probes which is scaled to the mean of data forms the vector of displacements $${\varvec{q}} = [\begin{array}{*{20}c} {u_{x} } & {v_{y} ]} \\ \end{array}^{{\text{T}}}$$ which is employed with Eqs. (16) and (18) to obtain the experimental potential energy and effective stiffness values. The resonance frequency of the experimental configuration is obtained for the crack-free case to be $\Omega_{\exp } \cong 57{\text{ Hz}}$ where the obtained one from the theoretical crack-free Jeffcott rotor model is $\Omega_{JF} \cong 59{\text{ Hz}}$.

**Table 1:** Physical parameters of the rotor.

Physical parameter description	Value
Length of the steel shaft $(L)$	700 mm
Radius of the steel shaft $(R)$	10 mm
Density of the steel shaft $(\rho_{s} )$	$7850{\text{ kg/m}}^{3}$
Modulus of elasticity of the steel shaft $(E)$	$2.1 \times 10^{11} {\text{ N/m}}^{2}$
Mass unbalance $(m \cdot e)$	$1 \times 10^{ - 4} {\text{ kg}} \cdot {\text{m}}$
Unbalance force angle $(\beta )$	varying
External viscous damping coefficient $(\gamma )$	$100{\text{ s}}^{ - 1}$
Internal viscous damping coefficient $(\zeta )$	$2 \times 10^{ - 7} {\text{ s}}$
Outer radius of the aluminum rigid disk $(r_{o} )$	150 mm
Inner radius of the aluminum rigid disk $(r_{i} )$	10 mm
Thickness of the aluminum rigid disk $(h)$	15 mm
Mass of the aluminum rigid disk	$0.663{\text{ kg}}$

### Steady-state response

The response of the cracked rotor system for steady-state operations is obtained from the numerical integration of Eq. (3) according to the given physical parameters in Table 1 of the considered cracked system. Therefore, the numerical and experimental whirl amplitudes are obtained for open and breathing crack models. Accordingly, Eqs. (16) and (18) are employed with the numerical and experimental whirl responses for calculating the potential energy and effective stiffness values.

#### Open crack model

**Figure 4:**
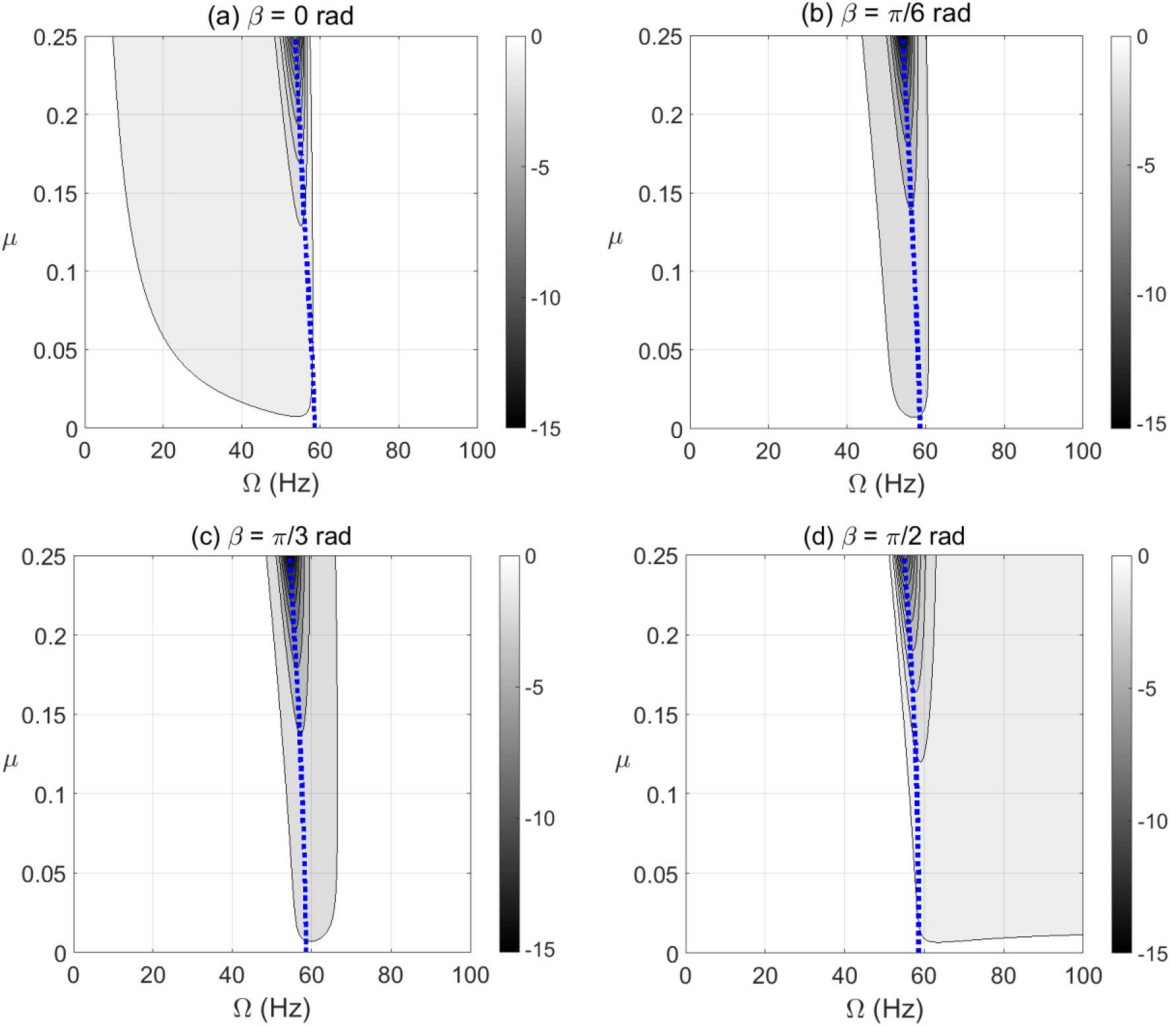
Effect of the open crack model depth $\mu$ at varying shaft rotational speeds on the potential energy content in steady-state response at unbalance force angles $\beta = 0{\text{ rad}}$ in (**a**), $\beta = \pi /6{\text{ rad}}$ in (**b**), $\beta = \pi /3{\text{ rad}}$ in (**c**) and $\beta = \pi /2{\text{ rad}}$ in (**d**).

**Figure 5:**
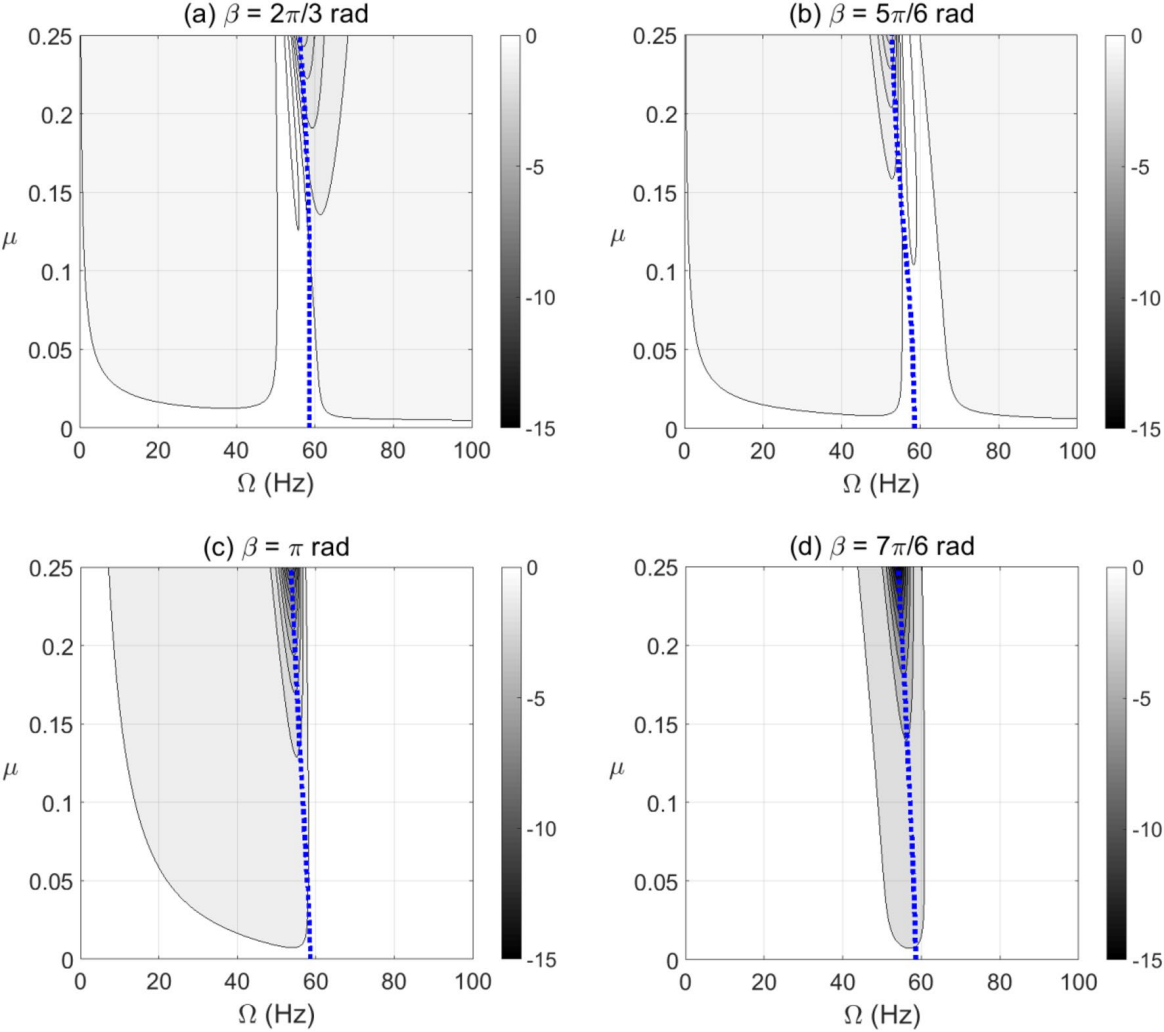
Effect of the open crack model depth $\mu$ at varying shaft rotational speeds on the potential energy content in steady-state response at unbalance force angles $\beta = 2\pi /3{\text{ rad}}$ in (**a**), $\beta = 5\pi /6{\text{ rad}}$ in (**b**), $\beta = \pi {\text{ rad}}$ in (**c**) and $\beta = 7\pi /6{\text{ rad}}$ in (**d**).

The unstable zones of shaft rotational speeds are usually dominated by negative potential and negative stiffness content. The positive potential energy values are set to zero in all contour plots in the paper to distinguish the negative potential energy zones. In addition, the blue dotted line in all contour plots represents the resonance forward whirl rotational speeds. Therefore, the effect of crack propagation on negative potential and effective stiffness during shaft operation at steady-state response is shown in Figs. 4 and 5 for different unbalance force vector orientations of gravity-free vertical rotor system. It was observed that the crack depth and unbalance force vector angles have significant impact on the extent and intensity of the negative potential energy zones. Extended zones of negative potential for wide range of rotational speeds are observed when the unbalance force angle is between $\beta = \pi /2{\text{ rad}}$ and $\beta = \pi {\text{ rad}}$ as shown in Fig. 5. Therefore, this zone of unbalance force vector angles significantly affects the stability of the cracked system and elevates the severity of crack propagation. Furthermore, it is also observed that at $\beta = \pi /6{\text{ rad}}$ and $\beta = \pi /3{\text{ rad}}$ in Fig. 4 and $\beta = 7\pi /6{\text{ rad}}$ in Fig. 5, the negative potential content has the highest levels at $\mu \ge 0.25$.

**Figure 6:**
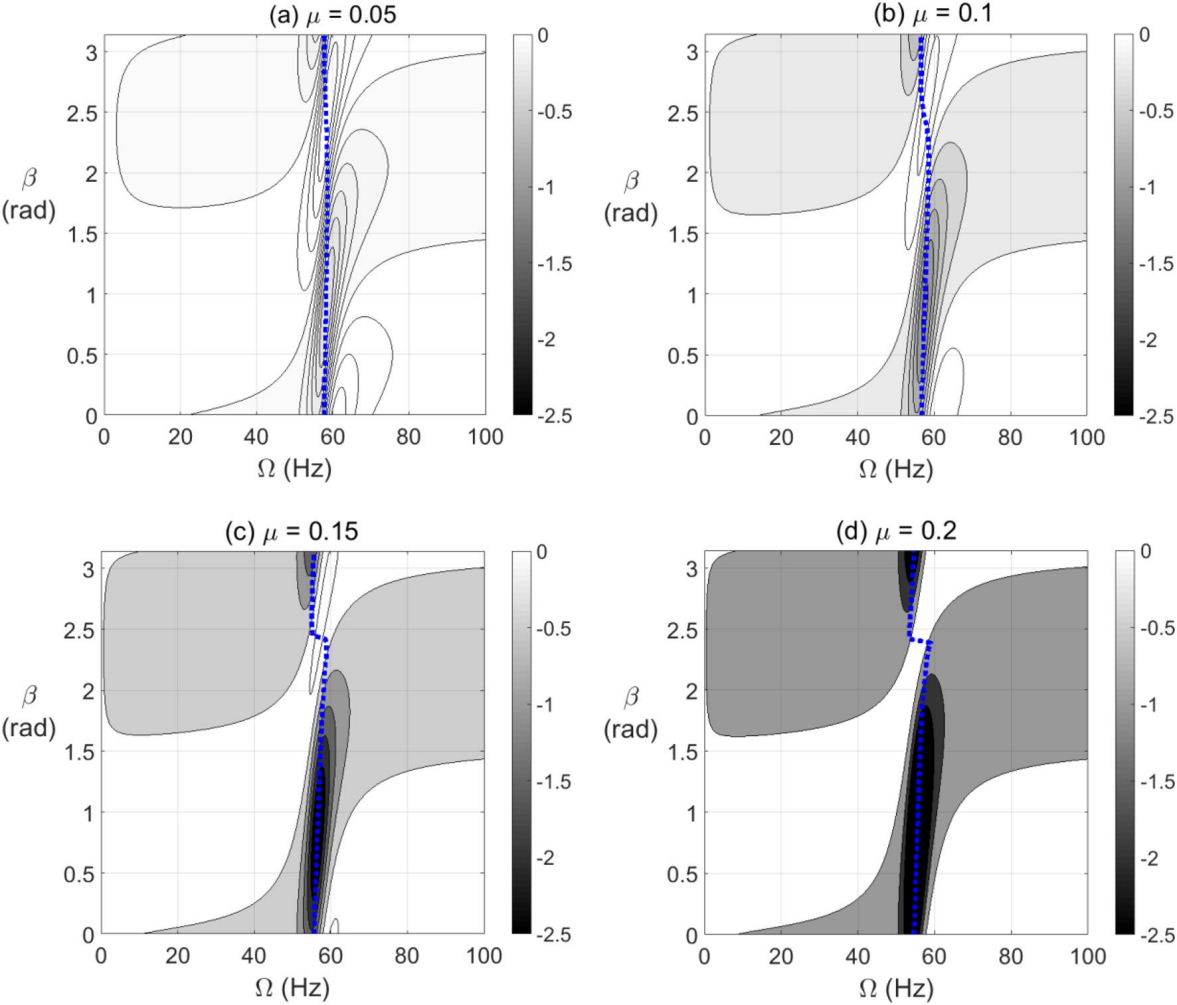
Effect of unbalance force vector angle $\beta$ at varying shaft rotational speeds on potential energy content in steady-state response of vertical cracked rotor system with open crack model depths $\mu = 0.05$ in (**a**), $\mu = 0.1$ in (**b**), $\mu = 0.15$ in (**c**) and $\mu = 0.2$ in (**d**).

**Figure 7:**
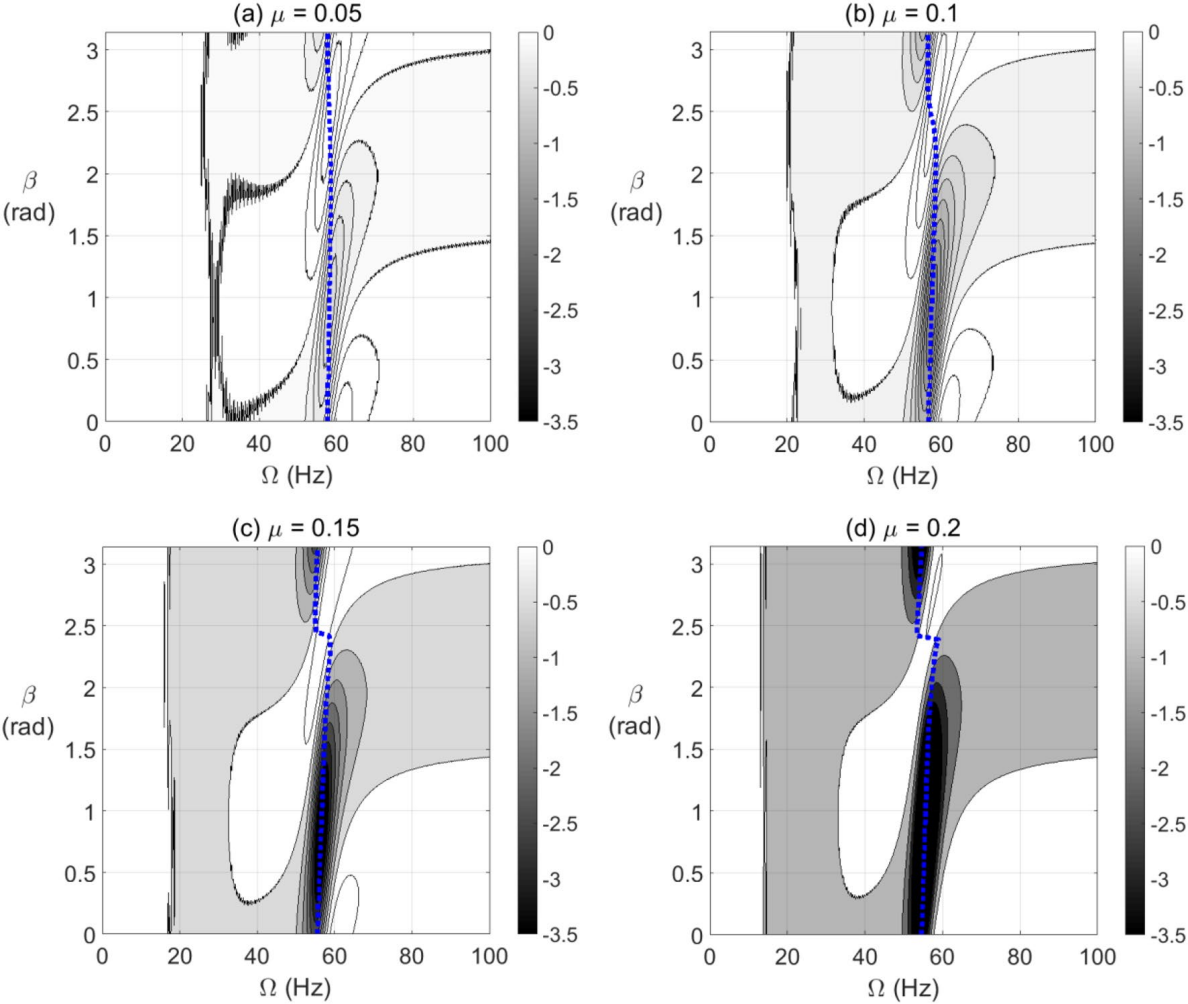
Effect of unbalance force vector angle $\beta$ at varying shaft rotational speeds on potential energy content in steady-state response of horizontal cracked rotor system with open crack model depths $\mu = 0.05$ in (**a**), $\mu = 0.1$ in (**b**), $\mu = 0.15$ in (**c**) and $\mu = 0.2$ in (**d**).

To clearly identify the effect of varying $\beta$ on the level of negative potential rather than the ranges of its extent, the results are plotted in Fig. 6 for vertical shaft and in Fig. 7 for horizontal shaft configurations, respectively, at different crack depths. It is observed in these figures that the zones of $\beta$ values of high levels of negative potential and stiffness are nearly centered at $\beta = \pi /4{\text{ rad}}$. Therefore, near to this angle the crack propagation has the highest effect on the stability of the cracked system than other angles. It is also observed that the gravity has less impact on the negative potential energy content than other parameters as observed in Fig. 6 of vertical system and Fig. 7 of horizontal system.

#### Breathing crack model

**Figure 8:**
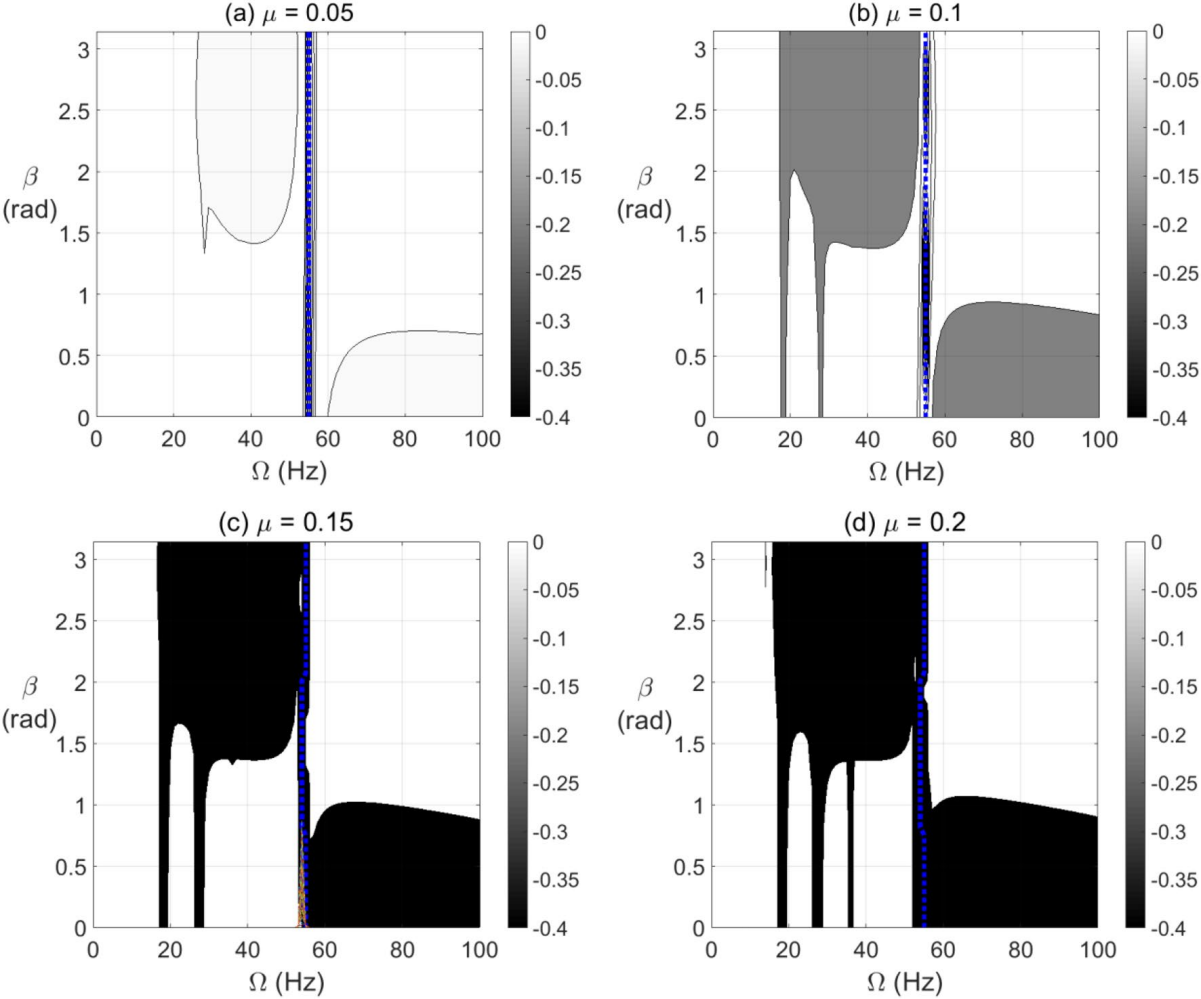
Effect of unbalance force vector angle $\beta$ at varying shaft rotational speeds on potential energy content in steady-state response of horizontal cracked rotor system with breathing crack model depths $\mu = 0.05$ in (**a**), $\mu = 0.1$ in (**b**), $\mu = 0.15$ in (**c**) and $\mu = 0.2$ in (**d**).

The effect of breathing crack model on negative potential energy and effective stiffness zones of the horizontal cracked rotor system for different crack depths, varying unbalance force vector angles and varying steady-state rotational speeds is shown in Fig. 8. Two major zones of negative potential and stiffness are observed at each crack depth where one of them appear in the pre-resonance zone at high values of $\beta$ and the other one appears in the post-resonance zone at low values of $\beta$. In addition other extended zones start to appear in the vicinity of 1/3, 1/2 and 2/3 of the resonance whirl rotational speed as shown. The intensity and recurrence of these zones are affected by the crack depth as shown. At $\mu = 0.2$, the level of negative potential energy and stiffness gets very high as shown from the gray-scale colored bar, especially in the neighborhood of the resonance rotational whirl speed. In addition, at resonance whirl rotational speed, the negative potential energy content is observed to be dominant. Both open and breathing crack models in Figs. 7 and 8 are associated with significant negative potential energy zones that appear at pre- and post-resonance rotational speeds. These zones are strongly affected by the crack depth and the unbalance force vector orientation.

#### Experimental results

**Figure 9:**
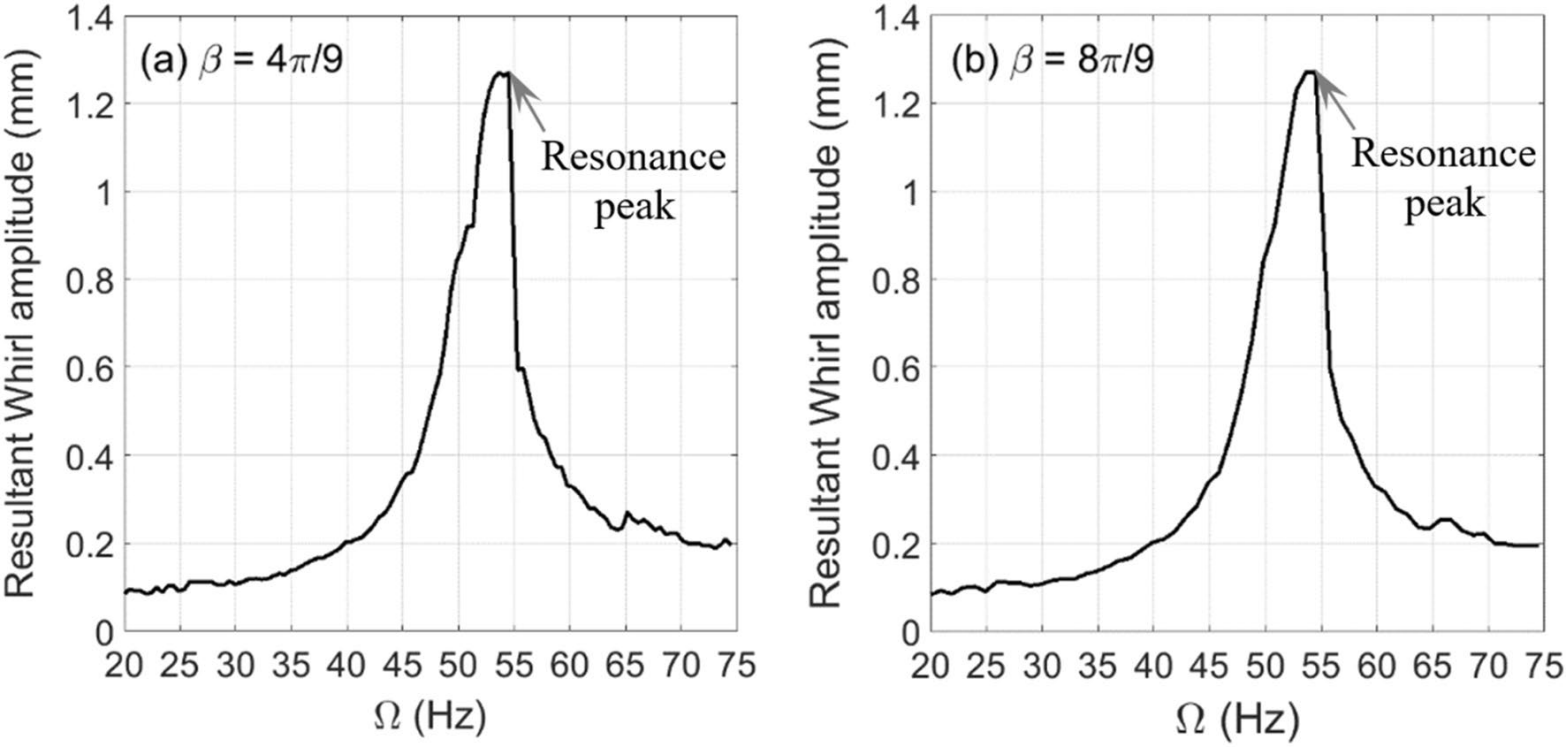
Experimental whirl amplitudes in steady-state response versus the shaft rotational speeds at $\mu = 0.2$ and $\beta = 4\pi /9{\text{ rad}}$ in (**a**) and $\beta = 8\pi /9{\text{ rad}}$ in (**b**) of the horizontal cracked rotor system.

**Figure 10:**
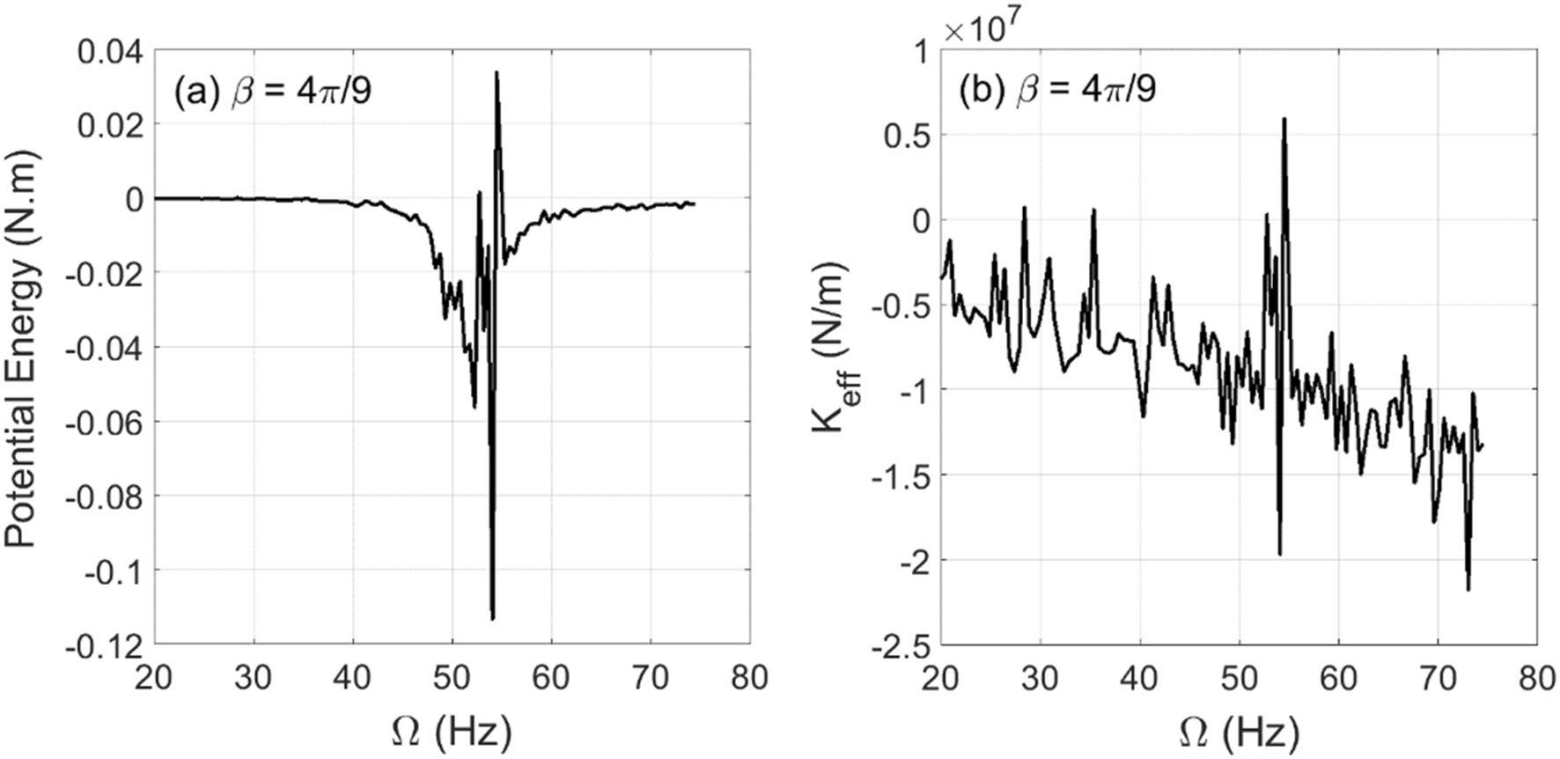
Experimental results in steady-state response of potential energy content in (**a**) and the corresponding effective stiffness content in (**b**) for varying shaft rotational speeds at $\mu = 0.2$ and $\alpha = 25{\text{ rad/s}}^{2}$ of the horizontal cracked rotor system.

**Figure 11:**
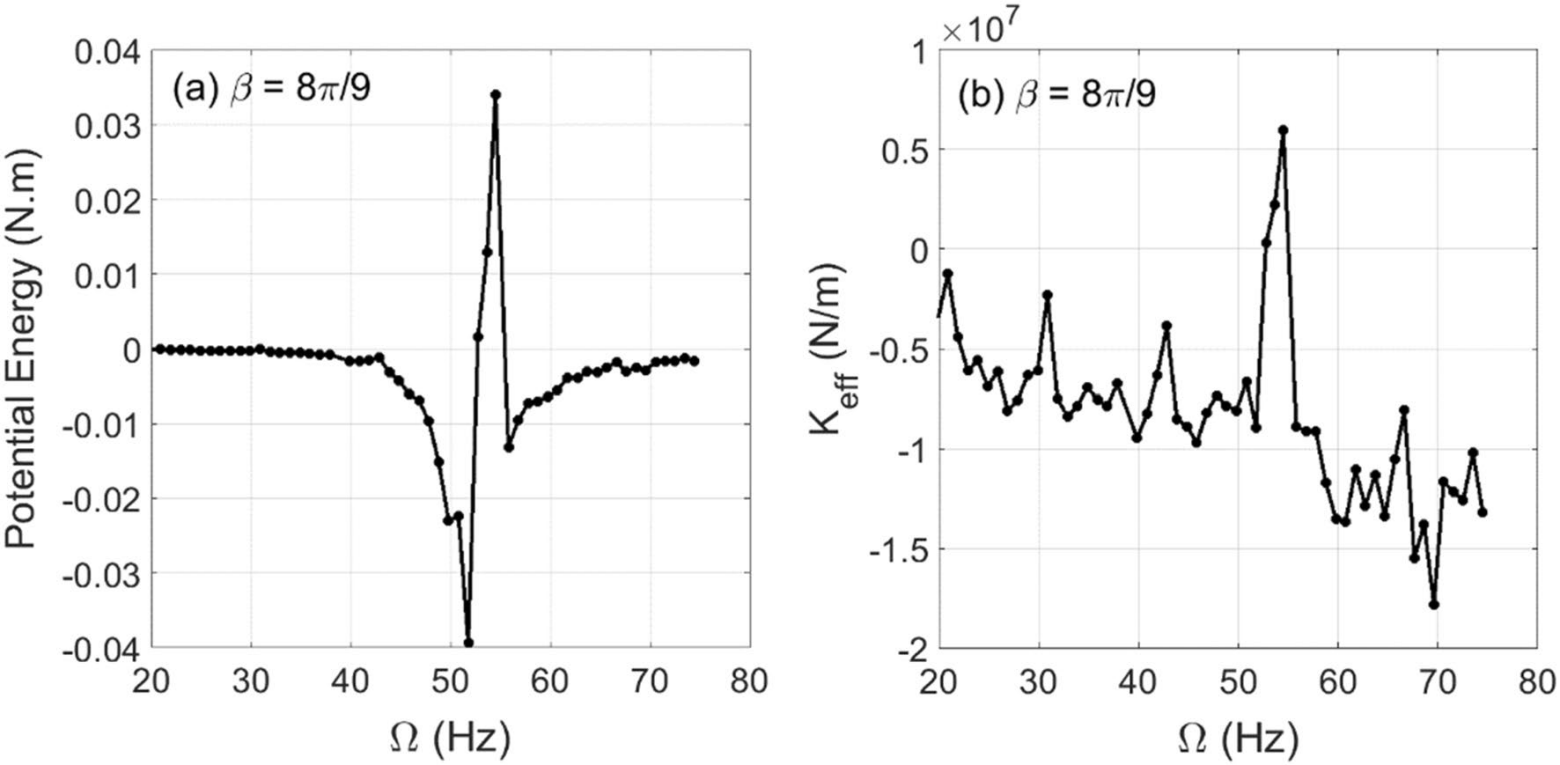
Experimental results in steady-state response of potential energy content in (**a**) and the corresponding effective stiffness content in (**b**) for varying shaft rotational speeds at $\mu = 0.2$ and $\alpha = 25{\text{ rad/s}}^{2}$ of the horizontal cracked rotor system.

The changes in experimental whirl amplitudes with respect to the shaft rotational speed are plotted in Fig. 9 for crack depth $\mu = 0.2$ at two different unbalance force vector angles $\beta = 4\pi /9{\text{ rad}}$ and $\beta = 8\pi /9{\text{ rad}}$. The resonance rotational speed is obtained from this figure for $\beta = 4\pi /9{\text{ rad}}$ and $\beta = 8\pi /9{\text{ rad}}$ at $\mu = 0.2$ to be $\Omega_{\exp } \cong 54.5{\text{ Hz}}$ where the crack-free one is $\Omega_{\exp } \cong 57{\text{ Hz}}$. The obtained potential energy and the corresponding effective stiffness values based on the experimental whirl amplitudes and Eqs. (16) and (18) are plotted in Fig. 10 for $\beta = 4\pi /9{\text{ rad}}$ and in Fig. 11 for $\beta = 8\pi /9{\text{ rad}}$. It is observed in these figures that in the vicinity of the critical rotational speed $\Omega_{\exp } \cong 54.5{\text{ Hz}}$ significant transitions between positive and negative effective stiffness values take place with various intensities. These observations are in good agreement with the numerical simulation prediction in Fig. 7d for both considered unbalance force angles.

### Transient response

The response of horizontal cracked rotor system for transient operations is obtained for Eq. (3) by numerical integration for both open and breathing crack models. Accordingly, the numerical and experimental whirl amplitudes and velocities are obtained for potential energy and effective stiffness calculations according to Eqs. (16) and (18).

#### Open crack model

**Figure 12:**
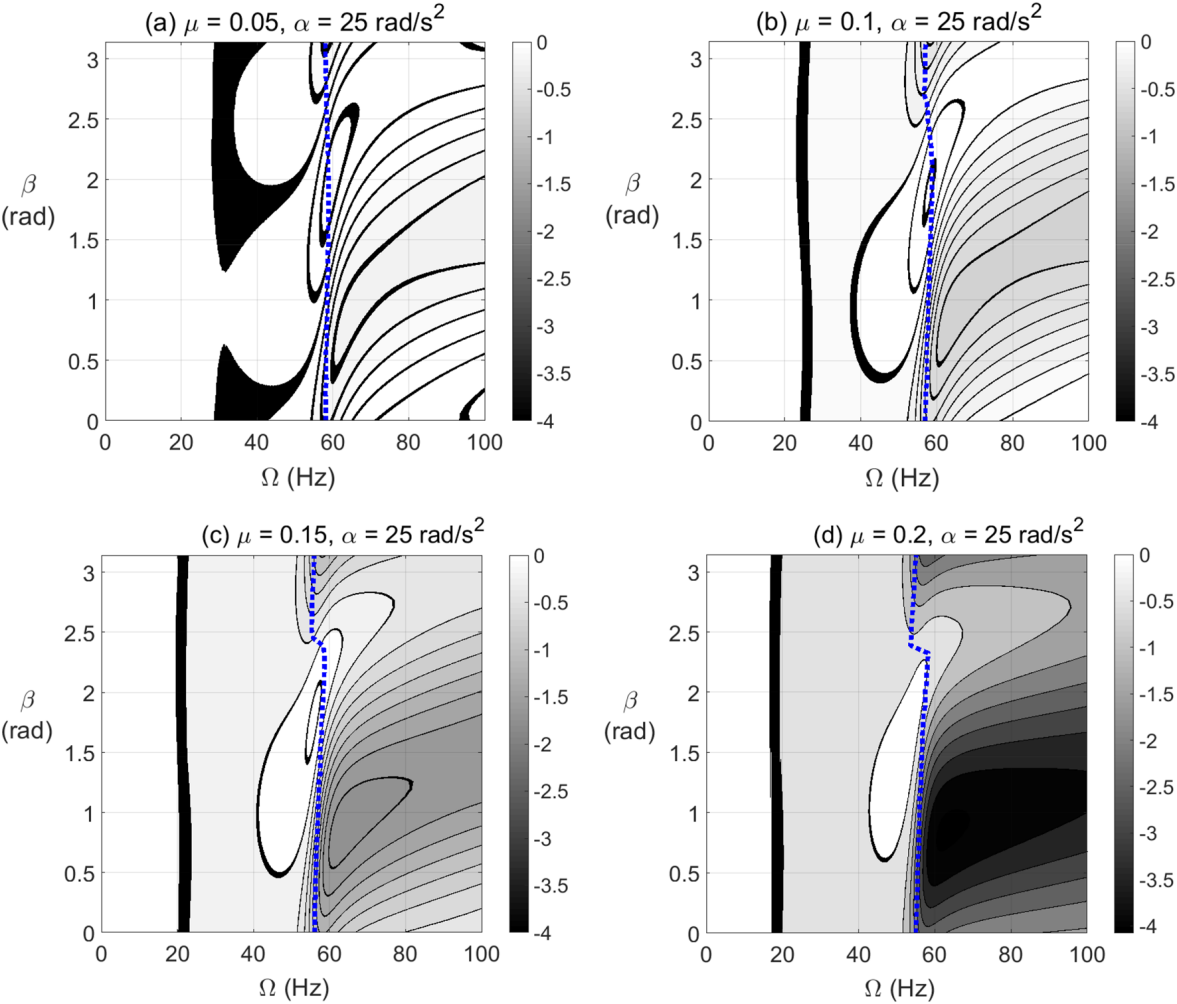
Effect of unbalance force vector angle $\beta$ at varying shaft rotational speeds for $\alpha = 25{\text{ rad/s}}^{2}$ on the potential energy content in transient response at open crack depths $\mu = 0.05$ in (**a**), $\mu = 0.1$ in (**b**), $\mu = 0.15$ in (**c**) and $\mu = 0.2$ in (**d**).

**Figure 13:**
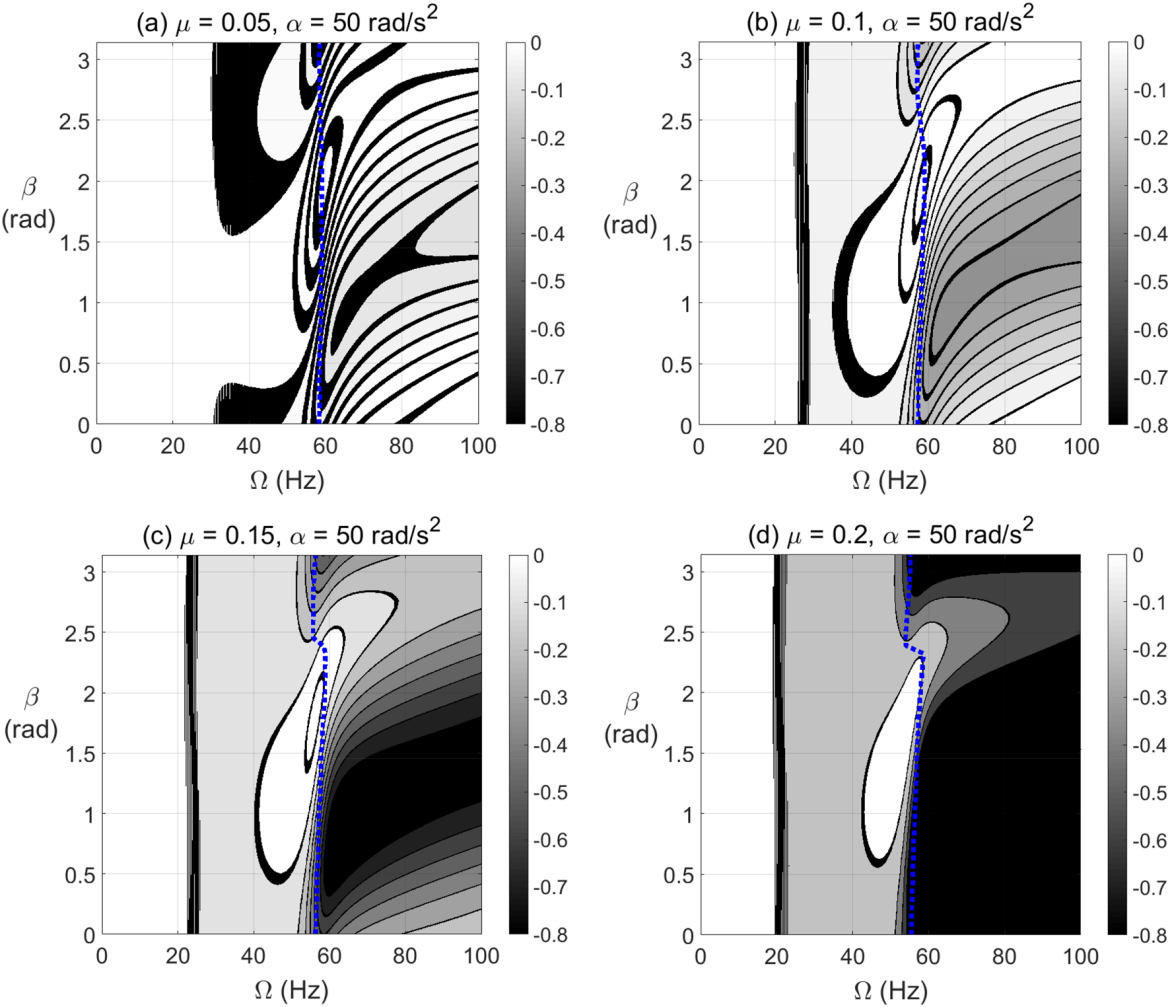
Effect of unbalance force angle $\beta$ at varying shaft rotational speeds for $\alpha = 50{\text{ rad/s}}^{2}$ on the potential energy content in transient response at open crack depths $\mu = 0.05$ in (**a**), $\mu = 0.1$ in (**b**), $\mu = 0.15$ in (**c**) and $\mu = 0.2$ in (**d**).

The effect of open crack propagation and unbalance force vector orientation on the negative potential energy and stiffness content in the transient response at which a passage through resonance rotational speed takes place is shown in Fig. 12 for $\alpha = 25{\text{ rad/s}}^{2}$ and in Fig. 13 for $\alpha = 50{\text{ rad/s}}^{2}$. It is observed that the crack depth and unbalance force vector angles have a significant impact on the extent and level of negative potential and stiffness zones. Wider zones of high negative potential energy are more observed at the post resonance rotational speeds rather than the pre-resonance rotational speeds. These highest levels of negative potential are observed near to $\beta = \pi /2{\text{ rad}}$ at $\alpha = 50{\text{ rad/s}}^{2}$ and significantly affected by crack depth propagation for both angular acceleration rates as shown from the gray-scale colored bar.

#### Breathing crack model

**Figure 14:**
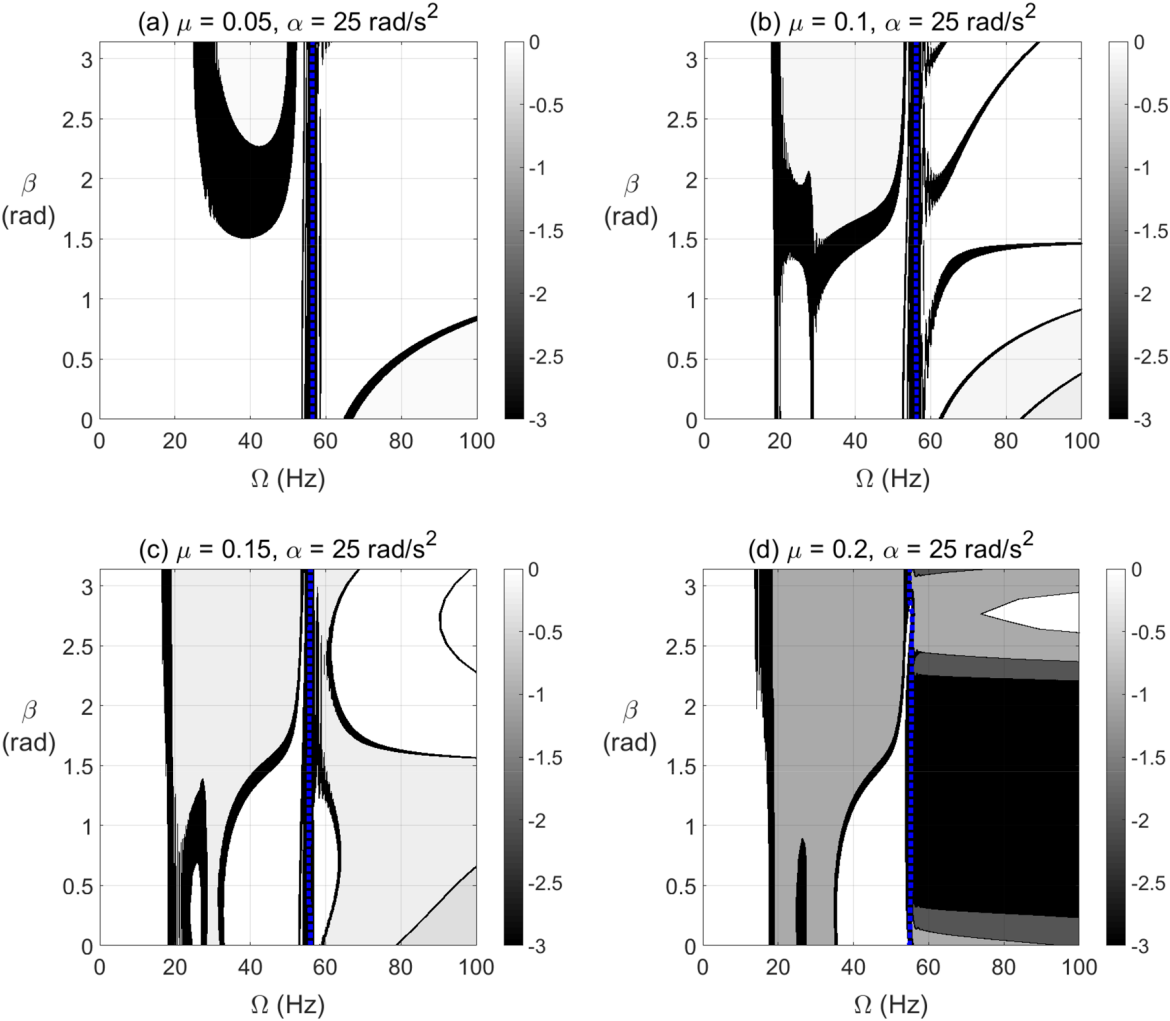
Effect of unbalance force vector angle $\beta$ at varying shaft rotational speeds for $\alpha = 25{\text{ rad/s}}^{2}$ on the potential energy content in transient response at breathing crack depths $\mu = 0.05$ in (**a**), $\mu = 0.1$ in (**b**), $\mu = 0.15$ in (**c**) and $\mu = 0.2$ in (**d**).

**Figure 15:**
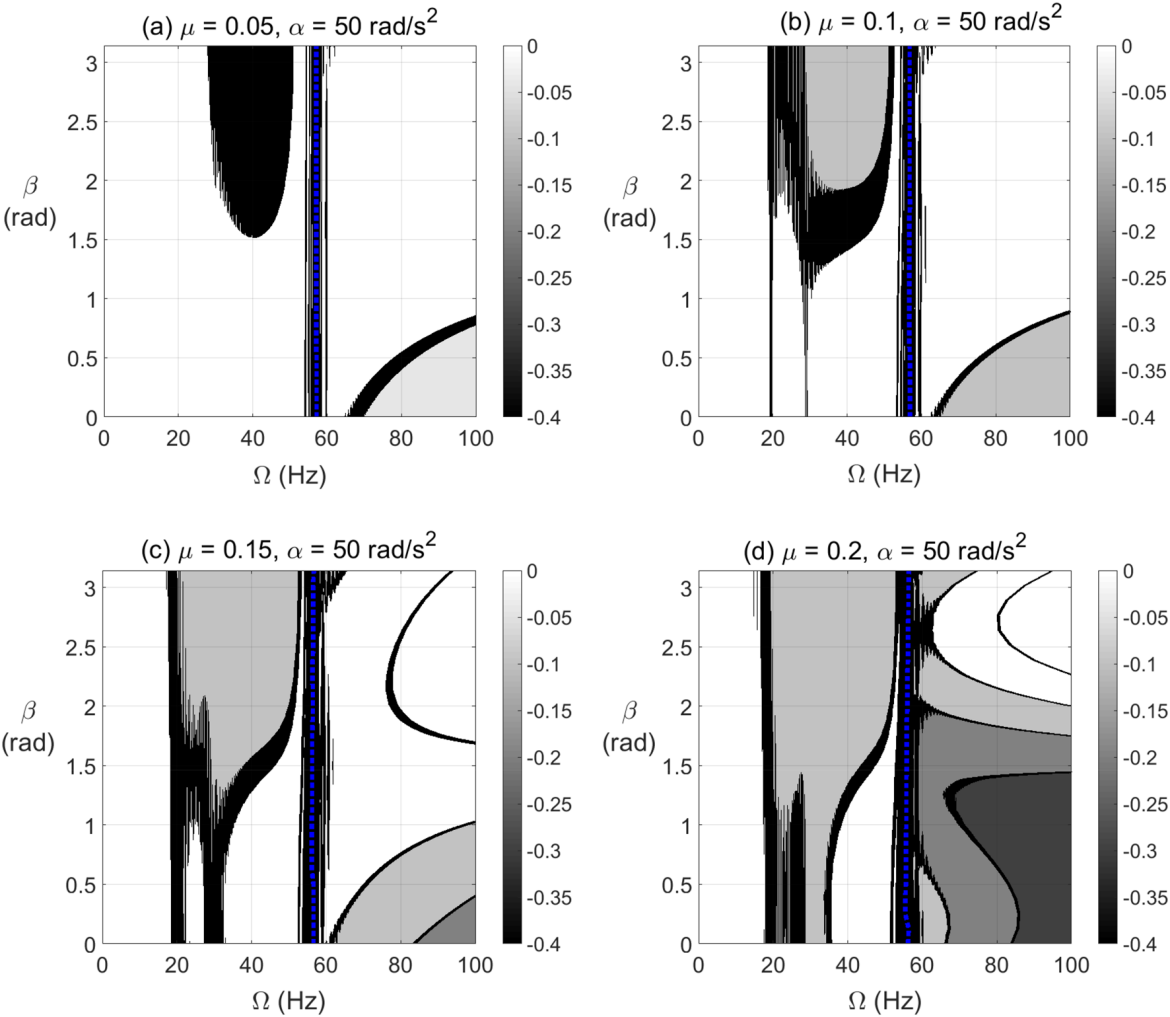
Effect of unbalance force vector angle $\beta$ at varying shaft rotational speeds for $\alpha = 50{\text{ rad/s}}^{2}$ on the potential energy content in transient response at breathing crack depths $\mu = 0.05$ in (**a**), $\mu = 0.1$ in (**b**), $\mu = 0.15$ in (**c**) and $\mu = 0.2$ in (**d**).

The effect of breathing crack propagation on negative potential and effective stiffness zones during shaft’s transient operation is shown in Figs. 14 and 15 at $\alpha = 25{\text{ rad/s}}^{2}$ and $\alpha = 50{\text{ rad/s}}^{2}$, respectively, for different crack depths and unbalance force vector orientations. It is clearly observed that the crack depth and unbalance force vector orientation have also a significant impact on the extent and intensity of the negative potential zones. Extended zones of negative potential for a wide range of rotational speeds are observed at higher crack depths. The behavior near 1/2, 1/3 and 2/3 of resonance rotational speed of the cracked system with breathing crack model in transient response in Figs. 14 and 15 is somehow close to that in Fig. 8 for steady-state response of similar rotor configuration with breathing crack model.

#### Experimental results

**Figure 16:**
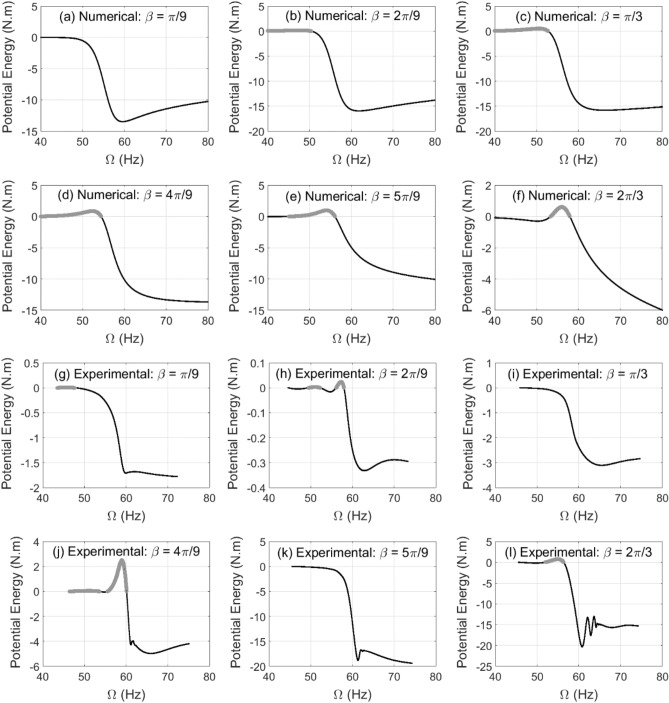
Numerical simulation results in (**a**)–(**f**) and the corresponding experimental results in (**g**)–(**l**) of the potential energy content in the transient whirl response at different unbalance force angles for $\alpha = 25{\text{ rad/s}}^{2}$ and $\mu = 0.2$(gray color for positive potential energy values).

To verify the numerical simulation findings of the accelerated shaft in transient response, both numerical and experimental whirl responses for horizontal and vertical whirl amplitudes are employed with Eqs. (16) and (18) for effective stiffness and potential energy calculations. Accordingly, the numerical simulation results of the potential energy content in Fig. 16 are compared with the experimental results for various values of unbalance force vector angles. It is observed that the zones of rotational speeds at which the potential energy content is of positive value precede the critical whirl speed zone in both numerical and experimental results for wide range of unbalance force vector angles. In addition, both numerical and experimental results show that the post-resonance zone of rotational speeds is dominated by negative potential energy content for all of the selected values of unbalance force vector angles.

In the shown range of rotational speeds at $\beta = \pi /9{\text{ rad}}$ in Fig. 16a, the numerical results only shows negative potential energy while their corresponding experimental results in Fig. 16g show a narrow zone of positive potential energy. Similarly, this is also observed at angles $\beta = \pi /3{\text{ rad}}$ in Fig. 16c,i and $\beta = 5\pi /9{\text{ rad}}$ in Fig. 16e,k. However, the remaining angles in Fig. 16 shows good agreement between the numerical and experimental results. This mismatch between some numerical and experimental results at some of the considered unbalance force angles does not affect the important conclusions stated in the previous paragraph.

## Remarks on findings

In the crack-free system, the negative stiffness content has not been captured in both steady-state and transient operations of the rotor system. However, in all of previous numerical and experimental results for steady-state and transient operations, it has been observed that the level of negative effective stiffness content is strongly affected by the transition from the pre-resonance to post-resonance zones of rotational speeds. The cracked shaft behaves like a buckled beam in its whirl orbit of rotation where the negative stiffness is generated due to the interaction between the time-periodic or time-varying stiffness content with the unbalance force excitation at steady-state and transient operations. It can be stated that during the bending of the rotating cracked shaft in its whirl orbit, the negative effective stiffness could indicate to that the shaft is more capable to further bending from its centerline. However, for positive effective stiffness the shaft seems to be stiffer for further bending from its centerline. Accordingly, the shaft during whirling could behave like a compressed static rod at both ends where bending from its centerline (buckling) takes place under the effect of the unbalance force excitation rather than end axial loads. Similar to the compressed rod, the shaft tends to resist bending toward its centerline and tends to be less stiff to bend away from the centerline. Accordingly, at the vicinity of resonance rotational speed, the negative stiffness content might result in severe vibration whirl amplitudes, leading to failure and complete damage as the crack keeps its propagation. The potential energy content can be investigated by using a well-established methodology in real life applications based on the instantaneous vibration data collected in rotor systems. For example, the vibration data in gas turbine units are collected at each bearing by horizontal and vertical accelerometers over long periods of running time. Therefore, these data can be processed to obtain the corresponding velocity and displacement responses for potential energy calculations. These potential energy calculations can be compared with reference calculations of an intact version of the rotor to observe any change that suggests cracks propagation.

## Conclusions

The Jeffcott rotor model is considered here to investigate the effect of interaction between the crack depth and the change in unbalance force vector orientation on potential energy and effective stiffness content in cracked rotor systems during transient or steady-state operations. The breathing and open crack models at steady-state and transient operations are considered in this study. The expression of the effective stiffness in the cracked rotor system has been obtained from direct integration of equations of motion. It has been found that there is a wide zones of rotational speeds and unbalance force vector angles at which high negative stiffness content appears in the numerical and experimental whirl responses of the considered crack rotor system. Therefore, the cracked rotor system in the vicinity of the resonance rotational speeds exhibits high levels of negative stiffness content where further crack propagation at several unbalance force vector angles put the system at high risk of rapid failure. The findings in this paper suggests analyzing the potential energy content in cracked rotors whirl response as a potential damage detection tool.

## Appendix

For breathing mechanism of the crack, the crack is assumed to be fully open position at $- \theta_{1} \le \theta \le \theta_{1}$ range, partially open at $\theta_{1} \le \theta < {{\left( {\pi + {\phi \mathord{\left/ {\vphantom {\phi 2}} \right. \kern-\nulldelimiterspace} 2}} \right)} \mathord{\left/ {\vphantom {{\left( {\pi + {\phi \mathord{\left/ {\vphantom {\phi 2}} \right. \kern-\nulldelimiterspace} 2}} \right)} 2}} \right. \kern-\nulldelimiterspace} 2}$ and $\pi - {\phi \mathord{\left/ {\vphantom {\phi 2}} \right. \kern-\nulldelimiterspace} 2} \le \theta < 2\pi - \theta_{1}$ ranges, and fully closed at ${{\left( {\pi + {\phi \mathord{\left/ {\vphantom {\phi 2}} \right. \kern-\nulldelimiterspace} 2}} \right)} \mathord{\left/ {\vphantom {{\left( {\pi + {\phi \mathord{\left/ {\vphantom {\phi 2}} \right. \kern-\nulldelimiterspace} 2}} \right)} 2}} \right. \kern-\nulldelimiterspace} 2} \le \theta \le {{\left( {3\pi - {\phi \mathord{\left/ {\vphantom {\phi 2}} \right. \kern-\nulldelimiterspace} 2}} \right)} \mathord{\left/ {\vphantom {{\left( {3\pi - {\phi \mathord{\left/ {\vphantom {\phi 2}} \right. \kern-\nulldelimiterspace} 2}} \right)} 2}} \right. \kern-\nulldelimiterspace} 2}$ where $\phi = \tan^{ - 1} \left( {s/b} \right)$ for the shown *s* and *b* dimensions in Fig. 2. Therefore, for the normalized crack depth $\mu$, the quantities in Eqs. (9) and (10) are calculated using the following equations^15,30^

19 $$ \begin{aligned} & I = \frac{\pi }{4}R^{4} \\ & \gamma = \left[ {\mu \left( {2 - \mu } \right)} \right]^{{{1 \mathord{\left/ {\vphantom {1 2}} \right. \kern-\nulldelimiterspace} 2}}} \\ & I_{x} = \frac{\pi }{8}R^{4} + \frac{{R^{4} }}{4}\left[ {\left( {1 - \mu } \right)\left( {2\mu^{2} - 4\mu + 1} \right)\gamma + \sin^{ - 1} \left( {1 - \mu } \right)} \right] \\ & I_{y} = \frac{\pi }{4}R^{4} - \frac{{R^{4} }}{12}\left[ {\left( {1 - \mu } \right)\left( {2\mu^{2} - 4\mu - 3} \right)\gamma + 3\sin^{ - 1} \left( \gamma \right)} \right] \\ & {\rm I}_{1} = {\rm I} - {\rm I}_{x}^{{}} \\ & {\rm I}_{2} = {\rm I} - {\rm I}_{y}^{{}} \\ & A_{1} = R^{2} \left[ {\pi - \cos^{ - 1} \left( {1 - \mu } \right) + \left( {1 - \mu } \right)\gamma } \right] \\ & e = \frac{{2R^{3} }}{{3A_{1} }}\left[ {\mu \left( {2 - \mu } \right)} \right]^{{{3 \mathord{\left/ {\vphantom {3 2}} \right. \kern-\nulldelimiterspace} 2}}} = \frac{{2R^{3} \gamma^{3} }}{{3A_{1} }} \\ & \overline{I}_{1} = I_{1} - A_{1} e^{2} \\ & \overline{I}_{2} = I_{2} \\ & \theta_{1} = \tan^{ - 1} \left( {\frac{{c + R\left( {1 - \mu } \right)}}{{R\sqrt {\mu \left( {2 - \mu } \right)} }}} \right) \\ & \theta_{2} = \frac{\pi }{2} + \cos^{ - 1} \left( {1 - \mu } \right) \\ \end{aligned} $$
